# Radius-Maumenee syndrome (idiopathic dilated episcleral vessels)

**DOI:** 10.3205/oc000247

**Published:** 2024-12-03

**Authors:** Carolina Tagliari Estacia, Aluisio Rosa Gameiro Filho, Izadora Bouzeid Estacia da Silveira, Martina Estacia Da Cas, Rodrigo Rosa Gameiro

**Affiliations:** 1 Hospital de Clínicas de Passo Fundo (HCPF), Rio Grande do Sul, Brazil; 2 Universidade Federal de São Paulo – Escola Paulista de Medicina (Unifesp-EPM), São Paulo, Brazil; 3 Universidade Federal de Ciências da Saúde de Porto Alegre (UFCSPA), Porto Alegre, Brazil; 4 Universidade de Passo Fundo (UPF), Passo Fundo, Brazil; 5 Universidade Federal do Paraná (UFPR), Curitiba, Brazil

**Keywords:** increased episcleral venous pressure, Radius-Maumenee syndrome, idiopathic dilated episcleral vessels, eye abnormalities, glaucoma open-angle, ocular physiological processes

## Abstract

**Purpose:**

Idiopathic elevated episcleral venous pressure (IEEVP) or Radius-Maumenee syndrome (RMS) is a rare disease without any identified underlying cause. An increasing episcleral venous pressure (EVP) leads to raised intraocular pressure (IOP) and consequently glaucomatous damage of the optic nerve. The objective of this paper is to report this rare condition as well as its clinical management.

**Observations:**

During cataract evaluation a 38-year-old female patient referred to redness in her right eye that had started more than 3 years before. The IOP was 22 mmHg in her right eye and 14 mmHg in her left eye, although she was already using Timolol drops. Biomicroscopy revealed diffusely engorged episcleral vessels, without any other relevant findings. Intracranial magnetic resonance imaging (MRI) was normal. For that reason, the diagnosis of RMS was established. After 3 months, the patient remains in use of Timolol and Latanoprost eye drops, with intraocular pressure within normal levels. She continues to be followed up on a regular outpatient basis.

**Conclusions and significance:**

RMS is a diagnosis of exclusion, based on clinical findings and imaging exams. Treatment includes eyes drops and surgery in refractory cases.

## Introduction

Idiopathic elevated episcleral venous pressure (IEEVP) has been first described in 1968 [1]. However, it was only in 1978 when the term Radius-Maumenee syndrome (RMS) was used for the first time. It is a rare clinical entity characterised by increased episcleral venous pressure (EVP) without any underlying cause that leads to increased intraocular pressure (IOP) and glaucomatous damage of the optic nerve [2]. 

Arteriovenous fistulas, such as carotid-cavernous fistula (CCF), are the most frequent cause of raised IOP due to increased EVP [3]. Despite there being several other causes of EVP, such as venous sinus thrombosis, Sturge-Weber syndrome, thyroid ophthalmopathy, or space-occupying lesions [4], [5], [6], its idiopathic form (IEEVP) is considerably rare [2] – it is a diagnosis of exclusion, which cannot be made without an in-depth investigation [6]. In this case, imaging exams were normal, so the diagnosis RMS was made.

 This manuscript describes one case of a patient, female, 38 years old, with unilateral ocular hypertension secondary to IEEVP treated successfully. 

## Case description

**Figure 1:**
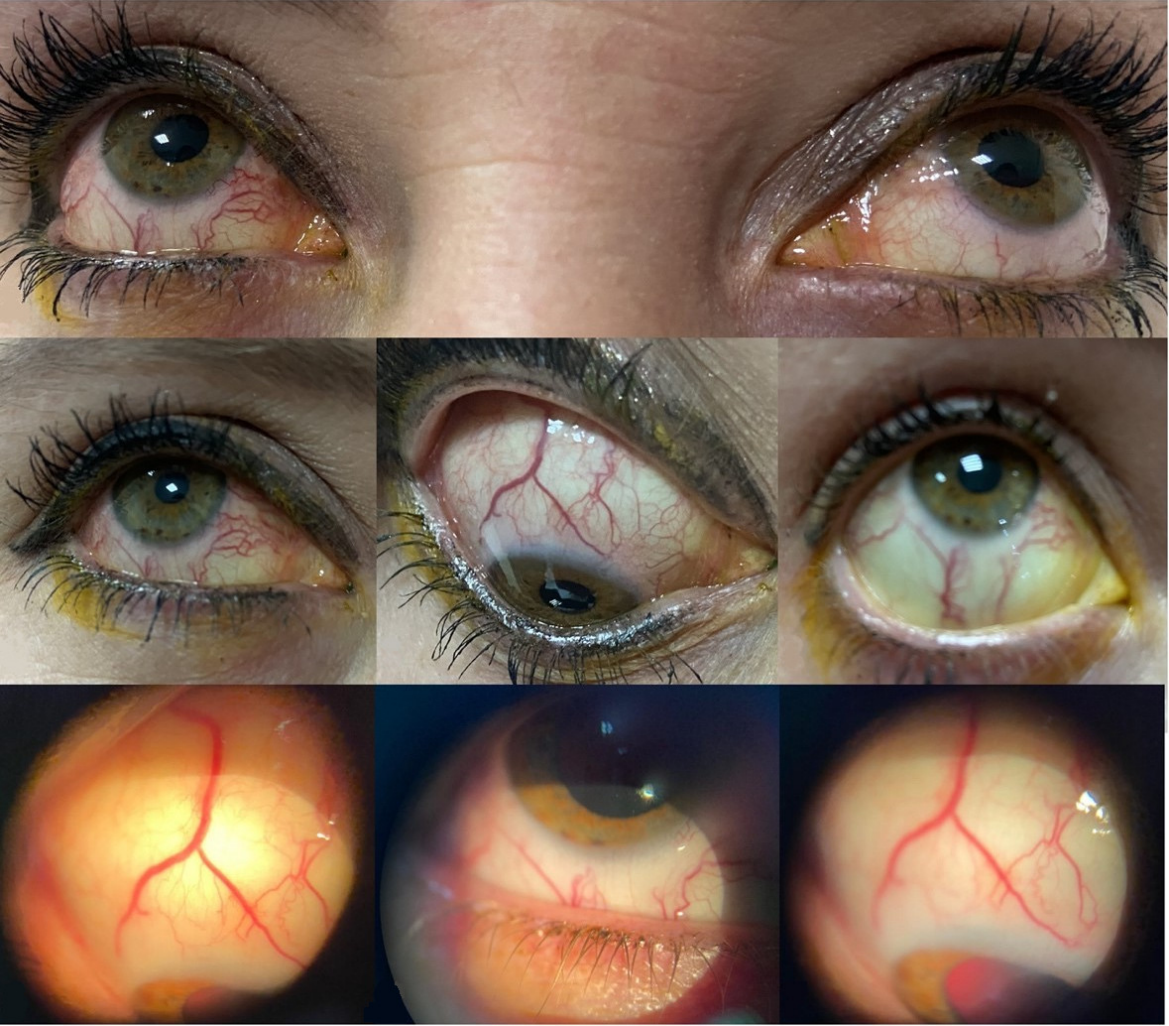
Diffusely enlarged and tortuous episcleral vessels in right eye

**Figure 2:**
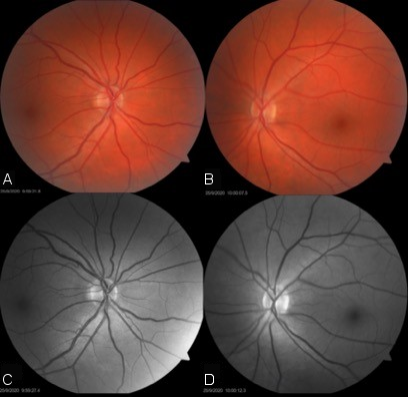
Retinography of the right eye (A and C) and left eye (B and D) with no abnormal findings

A 38-year-old female patient from southern Rio Grande do Sul state (Brazil) attended our office for evaluation of a cataract in her left eye. She denied any systemic diseases and referred to redness in her right eye that had started more than 3 years before, for which she was using Timolol drops. On ophthalmologic examination, her best-corrected visual acuity (BCVA) was 20/20 and 20/50 in the right and left eye, respectively. Biomicroscopy of the right eye showed diffusely engorged episcleral vessels (Figure 1 (Fig. 1)) without any other relevant findings; in her left eye, a cerulean cataract was evident. The intraocular pressure (IOP) was 22 mmHg in her right eye and 14 mmHg in her left eye. Fundoscopy was completely normal (Figure 2 (Fig. 2)), with a bilateral physiological excavation (0.3x0.3), and gonioscopy revealed an open-angle bilaterally. Findings of the adnexa were unremarkable, and the ocular motility was preserved, as well as pupillary reflexes. 

Campimetry and optic coherence tomography (OCT) did not show any relevant findings. An intracranial magnetic resonance imaging (MRI) angiogram was ordered to rule out cavernous carotid fistula, hemangiomas, and orbital alterations, which was completely normal. Thyroid tests were performed, all of them within normal values. Also, an angiography was performed, with normal results. In this context, in a patient with no intraorbital or intracranial findings that could explain a unilateral increase in intraocular pressure and the tortuous episcleral vessels, the diagnosis of RMS was established. After 3 months, the patient remains in use of Timolol and Latanoprost eye drops, with intraocular pressure within normal levels. She continues to be followed up on a regular outpatient basis. 

## Discussion

When the aqueous humor reaches the episcleral venous plexus, it flows into the anterior ciliary vein and subsequently to the superior ophthalmic veins. Then, it flows into the cavernous sinus, internal jugular vein, superior vena cava, and finally to the right atrium. Obstructions in this flow can cause elevations on the episcleral venous pressure, as well as dilations of the same. Elevated episcleral venous pressure (EVP) is an infrequent condition that is often associated with elevated intraocular pressure (IOP). The main cause of raised IOP due to increased EVP are arteriovenous malformations such as fistulas. Other possible causes are retrobulbar tumor, superior vena cava syndrome, thyroid eye disease, Sturge-Weber syndrome, and orbital varix [5], [6], [7], [8], [9]. 

Its idiopathic manifestation can be referred to as IEEVP and has been first described in 1968 by Minas and Podos with two cases of elevated EVP and blood in Schlemm’s canal [1]. Nonetheless, because in 1978 Radius and Maumenee reported 4 cases of idiopathic dilated episcleral vessels and glaucoma, IEEVP is also referred to as Radius-Maumenee syndrome [2]. 

The Radius-Maumenee syndrome can be understood as a disorder of undetermined etiology that courses with dilatation of episcleral vessels, EVP, and increase of IOP, commonly associated with glaucoma [6], [10], [11], [12], [13]. Pathologically, an increase of episcleral venous pressure beyond its normal range (8 to 10 mmHg) could halt regular aqueous outflow and, therefore, lead to elevated IOP [7]. Still, although hypotheses such as congenital vascular abnormalities, genetic predisposition, and hyalinization of the Schlemm’s canal have been proposed, the pathogenesis is still unclear [2], [6], [7], [10], [11], [14].

The reported epidemiology of RMS shows that it seems to have no gender predilection [6]. In addition to that, the age of onset ranges from teenage years to approximately the age of 70, with the large majority of cases appearing to course unilaterally or bilaterally with asymmetric involvement [5], [6], [8], [11], [12], [13], [15], [16], [17].

RMS is considered to be a rare condition: to the best of our knowledge, there have only been approximately 60 cases of IEEVP reported in the literature, as shown on our PubMed research for the terms “idiopathic elevated EVP”, “idiopathic dilated episcleral veins” and “Radius-Maumenee syndrome” (05/29/2022). The terms were chosen according to a similar approach by Sun et al. [7].

The most common clinical signs for this syndrome are elevated IOP with corresponding optic nerve and visual field findings consistent with glaucoma and dilated episcleral veins. Also, an open angle with blood in Schlemm’s canal can be observed by gonioscopy [7]. There are available methods for EVP measurement, but they remain restricted for research purposes [6], [12], [18], [19].

The diagnosis of RMS is obtained by exclusion, hence, it is necessary to dismiss other possible causes of increased EVP [6], [7], [10], [17]. Differential diagnosis includes carotid-cavernous-sinus fistula, Sturge-Weber syndrome, cavernous sinus thrombosis, thyroid related ophthalmopathy, superior vena cava syndrome, tumors, and scleritis. Image exams, such as cerebral angiography or doppler ultrasound, as well as systemic exams, such as thyroid testing must be performed in all patients to exclude these other possible causes. 

Due to the scarcity of cases and also to the fact that the diagnosis of RMS is clinical and based on exclusion, this condition can be easily neglected and misdiagnosed with other conditions. Previous reports show patients erroneously treated for chronic conjunctivitis, a more common abnormality in outpatient clinics, for a long time before the correct diagnosis of RMS was performed [17]. For this reason, RMS must be considered in patients presenting ocular hypertension and dilatation of episcleral vessels [10]. Also, episcleral vessel dilation in non-inflamed eyes should always be a red flag to the ophthalmologist for possible glaucoma [8]. It is known that a delay in establishing the diagnosis can course with negative impacts on the patient’s vision [8], [20]. 

The treatment for RMS is often challenging. Generally, the first approach consists in topical medications for glaucoma [7], [8], [17]. Still, in most cases, the necessary doses are extremely high, and the majority of patients end up needing other measures after a while [5], [6], [7]. In those scenarios – continued progression on maximum-tolerated pharmacological therapy – glaucoma surgery is the best call [7], [8]. According to the literature, several cases of IEEVP reported were managed with procedures including trabeculectomy, penetrating cyclodiathermy, and sinusotomy, many with favorable outcomes [7].

## Conclusion

RMS is a rare syndrome, which courses with increased IOP and engorged episcleral vessels. Due to the fact that it is an exclusion diagnosis, it is necessary to rule out other more common conditions, including some life-threatening causes of increased EVP. Currently, there are no objective techniques for measuring EVP in ophthalmological daily routine. Patients should be managed as open-angle glaucoma, with topical medication. Furthermore, in some cases, filtrating surgeries will be necessary. This case highlights the importance of suspecting of RMS in patients with long-standing eye redness.

## Notes

### Authors’ ORCIDs

- Carolina Tagliari Estacia: 0000-0001-8186-4975
- Aluisio Rosa Gameiro Filho: 0000-0002-8787-0417
- Izadora Bouzeid Estacia da Silveira: 0000-0003-4792-9733
- Martina Estacia Da Cas: 0000-0003-0753-0752
- Rodrigo Rosa Gameiro: 0000-0001-9600-6228

### Patient consent

The patient has provided written informed consent.

### Competing interests

The authors declare that they have no competing interests.
